# Resolving the Phylogenetic Placement of the FSfaCV/PCV5‐Related Viruses Within the Genus *Macochavirus*

**DOI:** 10.1155/tbed/8481347

**Published:** 2026-08-14

**Authors:** Wenqiang Wang, Liping Jiang, Tingting Niu, Miaojie Zhang, Lulu Chen, Xiaoye Jia, Lin Yuan, Kegong Tian, Xiangdong Li

**Affiliations:** ^1^ Jiangsu Co-innovation Center for Prevention and Control of Important Animal Infectious Diseases and Zoonoses, College of Veterinary Medicine, Yangzhou University, Yangzhou 225009, China, yzu.edu.cn; ^2^ Beijing Sino-science Gene Technology Co., Ltd., Beijing 102629, China; ^3^ National Research Center for Veterinary Medicine, Luoyang 471000, China; ^4^ Jiangsu Key Laboratory of Zoonosis, Jiangsu Interdisciplinary Center for Zoonoses and Biosafety, Yangzhou University, Yangzhou 225009, China, yzu.edu.cn

**Keywords:** cressdnavirus, high-throughput metagenomic sequencing, Pecoviridae, porcine *Macochavirus*, virus taxonomy

## Abstract

Metagenomic analysis of fecal samples from diarrheic pigs in China identified 10 complete circular single‐stranded DNA viral genomes previously designated as putative porcine circovirus 5 (PCV5)–like viruses. Genome characterization revealed a typical cressdnavirus organization with bidirectionally oriented Rep and Cap genes. Phylogenetic analyses based on complete genomes and encoded proteins showed that these viruses do not cluster with members of Circoviridae but instead form a distinct lineage within the family Pecoviridae, closely related to the genus *Macochavirus*. Sequence identity, genetic distance, and nucleotide diversity analyses supported their classification as a coherent viral group. Comparative analyses indicated greater sequence divergence in the Rep region than in the Cap region. These findings support reclassification of putative PCV5 as a porcine‐associated lineage within the genus *Macochavirus*, designated porcine *Macochavirus* (PMV), thereby resolving their long‐standing taxonomic ambiguity and expanding the recognized diversity of cressdnaviruses within the family Pecoviridae.

## 1. Introduction

High‐throughput metagenomic sequencing has substantially advanced virus discovery by enabling the unbiased detection of viral nucleic acids from diverse biological and environmental samples [1]. Unlike conventional diagnostic approaches that rely on prior knowledge of target pathogens, mNGS enables the simultaneous detection of diverse microbial genomes within a sample. This approach has proven particularly valuable in the investigation of complex infections and outbreaks caused by previously unknown or unexpected pathogens. By enabling the unbiased detection of both known and previously unrecognized viruses, metagenomic sequencing has greatly expanded our understanding of viral diversity across a wide range of hosts and ecological environments [2].

Circular replication‐associated protein‐encoding single‐stranded DNA viruses (cressdnaviruses) represent a highly diverse group of small circular DNA viruses characterized by compact genomes that typically encode a conserved replication‐associated protein (Rep) and a capsid protein (Cap) [3]. Members of this viral group have been detected in a broad range of hosts, including vertebrates, invertebrates, plants, and various environmental niches [4, 5]. Their widespread distribution across diverse biological systems highlights the remarkable evolutionary plasticity of cressdnaviruses and suggests a potential capacity for cross‐species transmission [6, 7]. With the rapid advancement of metagenomic sequencing technologies, the number of identified cressdnaviruses has increased dramatically, particularly in vertebrate hosts such as swine, revealing an unexpectedly complex evolutionary landscape [8–10].

In swine, several cressdnaviruses have been identified, among which porcine circoviruses (PCVs) are the most extensively studied. PCVs belong to the family Circoviridae and currently include four recognized species (PCV1–PCV4) [11–14]. Among them, PCV2 is a well‐established pathogen associated with a range of economically significant diseases in pigs [15]. In addition to these classical circoviruses, metagenomic investigations have revealed numerous circovirus‐like genomes in porcine samples. One such virus, provisionally designated PCV5, was initially identified because of its limited sequence similarity to known circoviruses [16]. However, its evolutionary origin and taxonomic position remain unresolved. Phylogenetic analyses have indicated that PCV5 is more closely related to fur seal feces‐associated circular DNA virus (FSfaCV) than to classical circoviruses, suggesting that these viruses may represent divergent cressdnavirus lineages distinct from the family Circoviridae [16]. Nevertheless, their precise evolutionary relationships and taxonomic placement remain unclear.

The taxonomic classification of these FSfaCV/PCV5‐related viruses has been further complicated by the limited availability of well‐annotated reference genomes and the lack of a clear taxonomic framework for these divergent cressdnaviruses. In addition, phylogenetic analyses based on single genes often produce inconsistent or weakly supported evolutionary signals [17]. Therefore, integrating multiple lines of evidence is essential for accurately resolving their evolutionary relationships and taxonomic positions.

In the present study, we identified 10 novel circular single‐stranded DNA viral genomes from diarrheic pigs in China using metagenomic sequencing. Through comprehensive analyses including genome characterization, phylogenetic reconstruction, sequence identity and genetic distance analyses, haplotype network inference, and genome‐wide nucleotide diversity profiling, we aimed to resolve their evolutionary relationships and clarify their taxonomic placement within the family Pecoviridae. Notably, our results provide evidence that these viruses, together with FSfaCV/PCV5‐related sequences, represent a distinct porcine‐associated lineage within the genus *Macochavirus*, thereby helping to resolve the long‐standing taxonomic ambiguity of these viruses.

## 2. Materials and Methods

### 2.1. Sample Processing, Nucleic Acid Extraction, and Metagenomic Sequencing Workflow

Samples were homogenized in sterile phosphate‐buffered saline (PBS) using a high‐frequency tissue homogenizer at 90 Hz for 90 s. The homogenates were then centrifuged at 3000 rpm for 5 min, and 0.2 mL of the supernatant was collected for downstream processing. A porcine kidney cell line (PK‐15) was included as a negative control throughout the experimental workflow.

Total nucleic acids (DNA and RNA) were co‐extracted from the supernatant using the MagMAX CORE Nucleic Acid Purification Kit (Thermo Fisher Scientific, USA) according to the manufacturer’s instructions based on a magnetic bead–based purification method. The concentrations of extracted nucleic acids were measured using the Equalbit 1× dsDNA HS Assay Kit and Equalbit RNA HS Assay Kit (Vazyme, China), respectively.

Sequencing libraries were prepared using the MGIEasy Fast Enzyme Fragmentation Library Prep Kit v2.0 (MGI Tech, China) following the manufacturer’s protocol. DNA nanoballs (DNBs) were generated using the MGISEQ‐2000RS high‐throughput rapid sequencing kit. High‐throughput sequencing was performed on the MGISEQ‐2000 platform with 100‐bp single‐end reads (SE100).

### 2.2. Bioinformatic Processing and Genome Assembly

Raw sequencing reads were initially processed using fastp (v0.23.2) with default parameters to perform adapter trimming and quality filtering [18]. The resulting clean reads were then aligned to the *Sus scrofa* reference genome (GenBank accession no. GCF_000003025.6) to remove host‐derived sequences using Bowtie2 (v2.3.5.1) under default parameters [19]. Reads that did not map to the host genome were retained for downstream analyses. The remaining non‐host reads were de novo assembled using SPAdes (v3.15.5) with default parameters [20]. The assembled contigs were subsequently compared against the NCBI nucleotide (nt) database using BLASTn (BLAST + v2.13.0). Based on sequence similarity searches, contigs showing significant similarity to cressdnaviruses were identified. Candidate circular viral genomes were identified based on the terminal sequence overlap and pseudo‐circularized by joining the genome termini. Clean reads were mapped back to the pseudo‐circularized genomes using Bowtie2, and genome circularity was evaluated by visual inspection of read coverage across the artificial junction using IGV. Continuous read support spanning the junction was considered evidence of a circular genome. Ten putative cressdnavirus genomes were obtained, with genome lengths ranging from 2881 to 2906 nt.

### 2.3. Phylogenetic Dataset Construction and Evolutionary Analyses

To determine the phylogenetic placement of the 10 viral genomes identified in this study, representative sequences from 21 families within the phylum Cressdnaviricota were included for analysis. Representative genomes from all currently recognized cressdnavirus families were retrieved from GenBank. Approximately four to six representative sequences were selected from each family, preferentially representing different genera to maximize taxonomic diversity. Representative sequences showing inconsistent phylogenetic placement relative to their assigned family were excluded from the final dataset. A total of 115 reference genomes were retrieved from GenBank, representing the families Bacilladnaviridae, Endolinaviridae, Vilyaviridae, Gandrviridae, Ouroboviridae, Draupnirviridae, Oomyviridae, Amesuviridae, Anicreviridae, Pecoviridae, Naryaviridae, Adamaviridae, Kirkoviridae, Kanorauviridae, Mahapunaviridae, Geplanaviridae, Circoviridae, Smacoviridae, Redondoviridae, Geminiviridae, and Genomoviridae. These reference sequences were combined with the 10 genomes generated in this study to construct the final dataset.

Prior to genome alignment, all complete circular genomes were standardized to start at the first nucleotide of the Rep gene to ensure a consistent sequence orientation for comparative analyses. Multiple sequence alignments were performed using MAFFT v7.505 with the automatic algorithm selection option enabled, which selects the most appropriate alignment strategy based on the sequence length and dataset size. Default parameters were used unless otherwise specified [21]. Phylogenetic analyses were conducted using IQ‑TREE v2.2.0 [22], with the best‑fit substitution model selected according to the Bayesian information criterion (BIC)—Q.pfam + F + R6 was identified as the optimal model for the initial dataset—and branch support assessed using 1000 ultrafast bootstrap (UFBoot) replicates [23]. To further resolve genus‑level classification, additional phylogenetic analyses were performed using sequences representing all eight recognized genera within the family Pecoviridae. Phylogenetic analyses were conducted based on three independent datasets (complete genome nucleotide sequences, Rep protein sequences, and Cap protein sequences). The best‑fit models selected by BIC were UNREST + FO + R4 for the complete genome dataset, LG + I+G4 for the Rep protein dataset, and LG + F+G4 for the Cap protein dataset. Final phylogenetic trees were visualized using FigTree v1.4.4, further annotated using iTOL, and the final layout was refined in Adobe Illustrator.

### 2.4. Pairwise Identity and Genetic Distance Analyses

To quantitatively assess the genome‐wide sequence similarity between the 10 viral genomes identified in this study and representative members of the eight recognized genera within the family Pecoviridae, pairwise sequence identity and genetic distance analyses were performed. The dataset comprised the 10 genomes generated in this study, 16 representative sequences from the eight recognized Pecoviridae genera, and seven previously reported FSfaCV/PCV5‐related sequences. Pairwise sequence identity analysis was conducted using the Sequence Demarcation Tool (SDT) v1.3. Complete genome sequences were first aligned using MAFFT v7.505 with default parameters, and pairwise nucleotide identity values were subsequently calculated in SDT based on the aligned sequences [24]. Genetic distances were calculated in MEGA v12 using the “Compute Pairwise Distances” function based on aligned complete genome sequences with default parameters [25].

### 2.5. Haplotype Network Construction and Sliding Window Analyses

To further investigate the genetic relationships among Pecoviridae sequences at the population level, haplotype network analysis was performed based on multiple sequence alignments of complete genome sequences. The aligned dataset generated using MAFFT v7.505 was imported into DnaSP v6 to identify haplotypes and generate haplotype data files [26]. To avoid potential biases introduced by ambiguous nucleotides (Ns), all alignment columns containing gaps, missing data, or ambiguous nucleotides were excluded prior to haplotype identification in DnaSP v6 [26]. The resulting data were exported in the NEXUS format for downstream analysis. Haplotype networks were constructed and visualized using PopART based on the Templeton–Crandall–Sing (TCS) algorithm [27]. The initial network layout was adjusted within PopART to improve the clarity, and the final graphical representation was further refined using Adobe Illustrator.

To evaluate genome‐wide patterns of nucleotide diversity, sliding window analyses of nucleotide diversity (*π*) were conducted using DnaSP v6 based on aligned complete genome sequences. The analysis was performed with a window size of 200 bp and a step size of 25 bp. The calculated *π* values were exported and visualized using R (v4.3.0).

## 3. Results

### 3.1. Identification and Genomic Characterization of Circular DNA Viruses in Diarrheic Pigs

**Figure 1:**
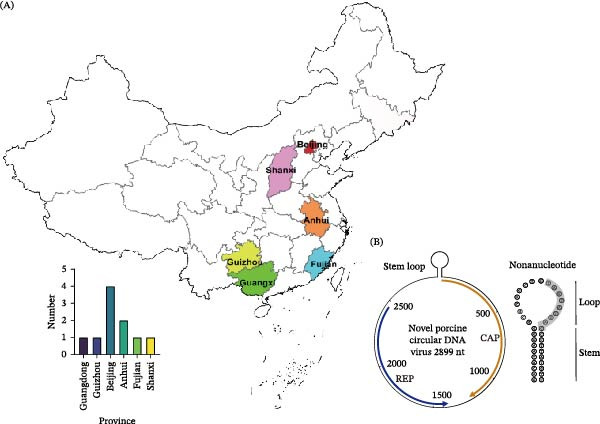
Genomic and geographic characterization of novel porcine circular DNA viruses. (A) Geographic distribution of the identified viral genomes. Colored regions indicate provinces where the sequences were detected, and the bar chart represents the number of sequences identified in each province. (B) Schematic diagram of the genomic structure of novel porcine circular DNA viruses.

A total of 105 fecal samples collected from pigs in different regions of China were subjected to metagenomic sequencing. Analysis of the sequencing data identified 10 complete circular viral genomes. These sequences were detected in samples originating from several provinces, including Guangdong (*n* = 1), Guizhou (*n* = 1), Beijing (*n* = 4), Anhui (*n* = 2), Fujian (*n* = 1), and Shanxi (*n* = 1) (Figure 1A). The genome sequences have been deposited in GenBank under accession numbers PX843008–PX843017.

The 10 viral genomes exhibited highly similar genomic features. The genome lengths ranged from 2881 to 2906 nt. All sequences contained the conserved nonanucleotide motif TAGTATTAC, located within the predicted stem–loop structure of the origin of replication, a characteristic feature of circular single‐stranded DNA viruses (Figure 1B). Genome annotation revealed two major open reading frames (ORFs) arranged in opposite orientations: a virion‐sense ORF encoding the capsid protein (Cap) and a complementary‐sense ORF encoding the replication‐associated protein (Rep). The Rep proteins contained conserved rolling‐circle replication motifs and helicase domains typical of cressdnaviruses. The predicted Rep and Cap genes consisted of approximately 1179–1182 and 1056 nucleotides, respectively. This bidirectional genome organization is commonly observed in cressdnaviruses.

BLAST analysis against the NCBI nucleotide database revealed that the 10 genomes shared the highest sequence similarity with previously reported FSfaCV/PCV5‐like viruses. However, the precise taxonomic placement of these viruses remains unclear. Therefore, further phylogenetic analyses were conducted to determine their evolutionary relationships and classification.

### 3.2. Phylogenetic Analysis Reveals That PCV5‐Like Viruses Belong to the Family Pecoviridae

To determine the taxonomic placement of the 10 viral genomes identified in this study, a phylogenetic analysis was performed using representative sequences from major families of Cressdnaviricota. A maximum‐likelihood phylogenetic tree was constructed based on the amino acid sequences of the Rep protein, including the 10 genomes obtained in this study and reference sequences retrieved from GenBank.

**Figure 2:**
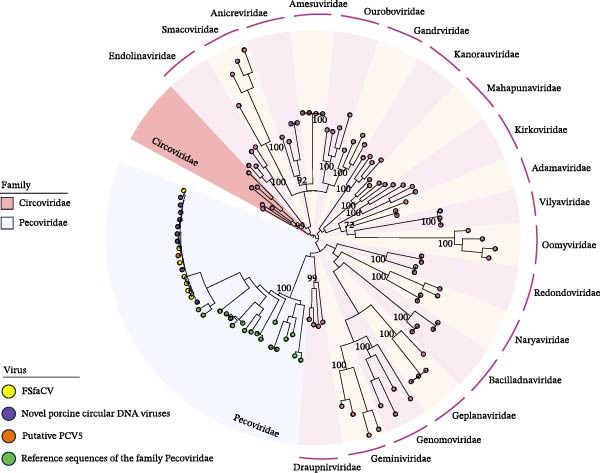
Maximum‐likelihood phylogenetic tree of cressdnaviruses inferred from Rep amino sequences. The tree was constructed using IQ‐TREE2 based on aligned Rep amino acid sequences. Representative sequences from 21 families of cressdnaviruses were included, together with previously reported FSfaCV and putative PCV5 sequences. The 10 new cressdnavirus sequences identified here are highlighted in purple, whereas sequences from other taxa are displayed in distinct colors. Colored blocks delineate different viral families, facilitating visualization of taxonomic classification and evolutionary relationships.

Phylogenetic analysis showed that the 10 newly identified viral genomes did not cluster with members of the family Circoviridae, including the known PCVs PCV1, PCV2, PCV3, and PCV4 (Figure 2). Instead, all 10 sequences were consistently grouped within the family Pecoviridae, forming a well‐supported monophyletic clade with a bootstrap value of 100.

Within the Pecoviridae clade, the 10 genomes clustered closely with previously reported FSfaCV/PCV5‐related sequences. This cluster was strongly supported by a bootstrap value of 100 and displayed relatively short branch lengths, indicating close genetic relationships among these viruses. These results demonstrate that the 10 viral genomes identified in this study represent additional members of the FSfaCV/PCV5 lineage within the family Pecoviridae.

### 3.3. Phylogenetic Analyses Place Porcine FSfaCV/PCV5‐Like Viruses Within the Genus *Macochavirus*

To further clarify the taxonomic placement of the 10 viral genomes within the family Pecoviridae, phylogenetic analyses were performed using representative sequences from the eight currently recognized genera of Pecoviridae: *Intivirus*, *Yaviravirus*, *Tambotovirus*, *Amaruvirus*, *Macochavirus*, *Cinchivirus*, *Quillavirus*, and *Capamivirus*, together with previously reported FSfaCV/PCV5 sequences.

**Figure 3:**
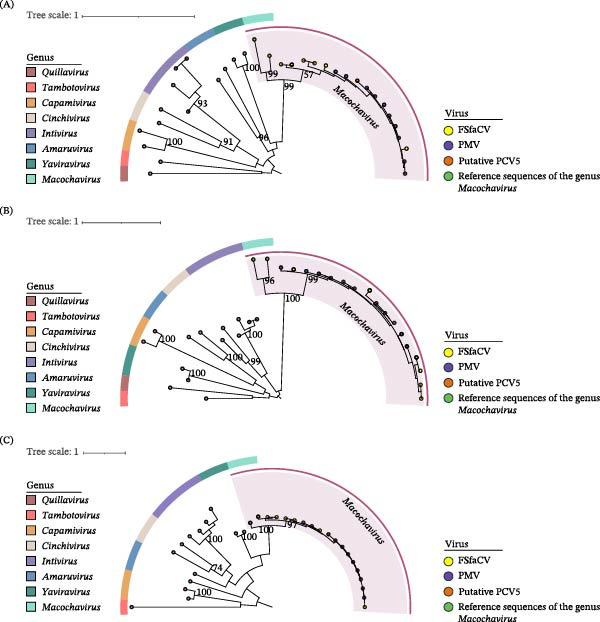
Phylogenetic analysis of PMV sequences within the family Pecoviridae. Maximum‐likelihood phylogenetic trees were inferred using IQ‐TREE2 based on (A) complete genome sequences, (B) Rep protein sequences, and (C) Cap protein sequences, respectively. The outer colored ring indicates different genera within the family Pecoviridae, while members of the genus *Macochavirus* are further distinguished using different colors to highlight intra‐genus variation.

A maximum‐likelihood phylogenetic tree based on complete genome sequences was constructed, showing that the 10 newly identified sequences clustered together with seven FSfaCV/PCV5‐related sequences (Figure 3A). This lineage was placed adjacent to representative members of the genus *Macochavirus* with strong bootstrap support (99), indicating that the 10 genomes are closely related to *Macochavirus*. However, the phylogenetic position of FSfaCV/PCV5 itself remains unresolved and may represent a separate genus within the family Pecoviridae.

Phylogenetic analysis of the Rep protein sequences showed that the 10 newly identified genomes clustered tightly with FSfaCV/PCV5‐related sequences, forming a strongly supported lineage (bootstrap = 99) placed within or adjacent to the *Macochavirus* clade (Figure 3B). The node supporting this placement received maximal bootstrap support (100), indicating that these viruses are closely related to the members of this genus but may represent a distinct lineage.

To further evaluate the evolutionary relationships, a phylogenetic analysis based on Cap protein sequences was performed. The 10 newly identified sequences clustered together with FSfaCV/PCV5‐related viruses and one representative sequence of the genus *Macochavirus*, forming a strongly supported lineage with a bootstrap value of 100 (Figure 3C). Notably, the other *Macochavirus* reference sequence formed a separate paraphyletic branch outside this cluster. Despite this separation, the overall relationship between FSfaCV/PCV5‐related viruses and members of the genus *Macochavirus* remained strongly supported. These results indicate that FSfaCV/PCV5‐related viruses are embedded within the evolutionary diversity of the genus *Macochavirus* rather than representing a distinct genus. Based on these findings, the viruses identified in this study together with FSfaCV/PCV5 are proposed to represent a porcine lineage within the genus *Macochavirus*, designated as porcine *Macochavirus* (PMV).

### 3.4. Sequence Identity and Genetic Distance Analyses Support the Proposed PMV Lineage

To further quantify the evolutionary relationships of the 10 newly identified porcine viral genomes with other members of the family Pecoviridae, pairwise sequence identity analysis was performed on the same dataset using the SDT.

**Figure 4:**
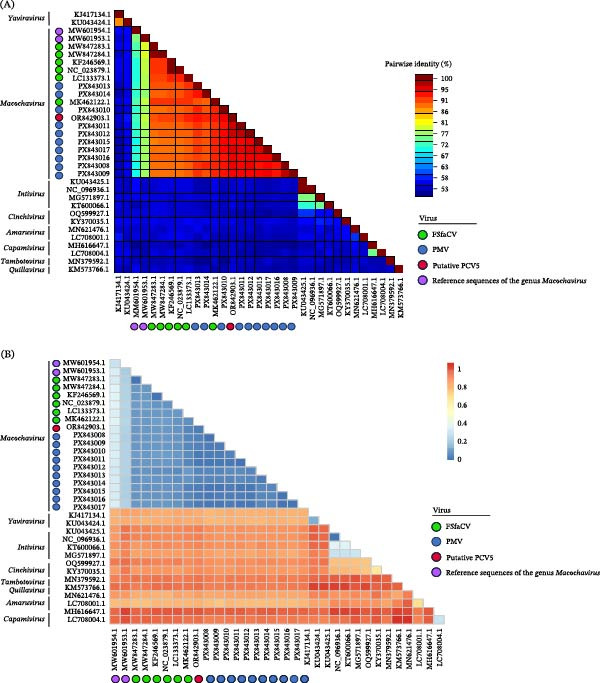
Sequence similarity and identity analysis of PMV sequences within the family Pecoviridae. (A) Sequence demarcation tool (SDT) analysis was conducted to evaluate the pairwise nucleotide identity between the 10 porcine‐derived cressdnaviruses (PMV) sequences identified in this study and representative members of different genera within the family Pecoviridae. (B) Sequence similarity analysis further illustrates the genetic relatedness among these viruses. The 10 PMV sequences are indicated by blue circles, and their corresponding genus assignments are labeled on the left.

The results showed that the 10 genomes formed a tight sequence similarity cluster with FSfaCV/PCV5‐related viruses, sharing pairwise nucleotide identities of 87% or higher (Figure 4A). These results are consistent with the phylogenetic analyses based on complete genome and Rep protein sequences, which placed these viruses within the same evolutionary lineage.

In comparison, the 17 genomes shared 76.3%–80.0% nucleotide identity with the type member of *Macochavirus uywa* (MW601953.1) and 69.5%–75.1% nucleotide identity with *Macochavirus kuci* (MW601954.1). In contrast, nucleotide identities with representatives of other genera within the family Pecoviridae were all below 56.1%, supporting the placement of the newly identified viruses within the genus *Macochavirus*. Genetic distance analysis produced results consistent with SDT analysis. The 17 genomes exhibited the lowest genetic distances among themselves (less than 0.10), moderate distances to the two *Macochavirus* reference sequences, with values ranging from 0.33 to 0.36 for MW601954.1 (*Macochavirus kuci*) and from 0.24 to 0.26 for MW601953.1 (*Macochavirus uywa*), and substantially larger distances (>0.77) to members of other genera (Figure 4B).

The relatively lower nucleotide identity and moderate genetic distances observed between the proposed PMV lineage and previously reported *Macochavirus* sequences suggest that PMV represents a genetically distinct lineage within the genus. Taken together, these quantitative analyses not only provide independent, quantitative support for the placement of FSfaCV/PCV5‐related viruses and the 10 newly identified genomes within the genus *Macochavirus*, but also highlight the expansion of the currently recognized diversity of *Macochavirus* through the addition of a novel porcine lineage, here designated PMV.

### 3.5. Haplotype Network Analysis of Pecoviridae Sequences Highlights the Proposed PMV Lineage

To further explore the genetic relationships among the newly identified porcine viruses and related members of the family Pecoviridae at the sequence population level, a haplotype network was constructed using complete genome sequences. Representative sequences were selected to cover the phylogenetic diversity of each genus, providing a framework for evaluating both intrageneric and intergeneric relationships.

**Figure 5:**
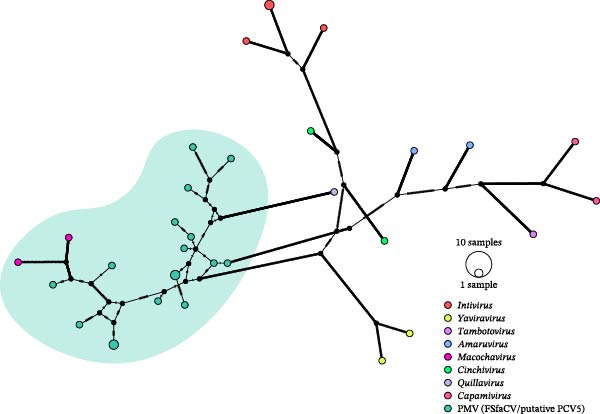
Haplotype network analysis of PMV sequences within the family Pecoviridae. The network was constructed based on aligned sequences using a TCS Network approach implemented in PopART and Launch DnaSP6 software. The 10 PMV sequences identified in this study, together with closely related FSfaCV and PCV5 sequences, are indicated by green circles. Each node represents a unique haplotype, and the connections between nodes indicate mutational steps among sequences. The network illustrates the haplotype structure and genetic relationships among these viruses.

The haplotype network showed that the 10 porcine viral genomes clustered with FSfaCV/PCV5‐related sequences, forming a compact haplotype group connected by a limited number of mutational steps (Figure 5). The two representative *Macochavirus* reference sequences were included within this cluster, but the distances between these reference sequences and the proposed PMV sequences were slightly greater than those among the PMV sequences themselves. Haplotypes representing other genera of Pecoviridae were separated from this cluster by multiple mutational steps, indicating clear intergeneric divergence.

These results demonstrate that the 10 newly identified porcine viral genomes cluster with FSfaCV/PCV5‐related sequences and form a distinct lineage within the genus *Macochavirus*, supporting their designation as PMV.

### 3.6. Genome‐Wide Nucleotide Diversity Patterns Support the Close Evolutionary Relationship Between PMV and *Macochavirus*

To examine genome‐wide evolutionary patterns and their concordance with phylogenetic inferences, a sliding‐window analysis of nucleotide diversity (*π*) was performed across complete genome sequences. The proposed PMV lineage, comprising the newly identified porcine viral genomes and FSfaCV/PCV5‐related sequences, was analyzed alongside representative sequences from each of the eight recognized genera of Pecoviridae.

**Figure 6:**
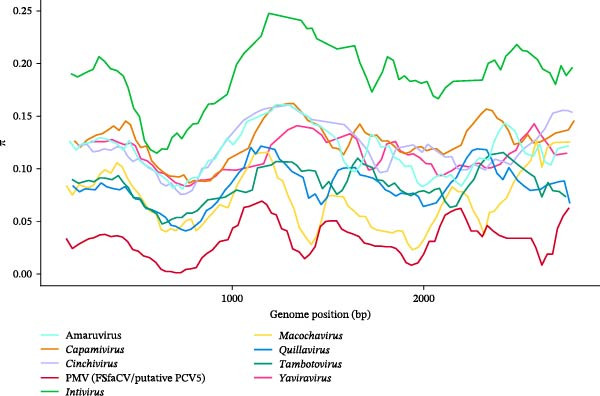
Sliding window analysis of nucleotide diversity (*π*) among PMV sequences and related viruses within the family Pecoviridae. The red line represents nucleotide diversity calculated from a combined dataset comprising the 10 PMV sequences together with closely related FSfaCV and PCV5 sequences. Other colored lines represent nucleotide diversity estimated from this combined dataset supplemented with representative sequences from each genus within Pecoviridae. This analysis highlights patterns of genetic variation across different sequence groupings.

Sliding‐window analyses generated a total of nine nucleotide diversity profiles, which were visualized as line plots across genome coordinates (Figure 6). The *π* profile calculated within the PMV lineage revealed relatively low and stable levels of nucleotide diversity across most genomic regions. When PMV sequences were analyzed together with representative sequences of different Pecoviridae genera, the diversity pattern observed in the comparison with representative *Macochavirus* sequences showed the most similar overall diversity levels and local fluctuation patterns to those observed within PMV. In contrast, diversity profiles obtained from analyses involving representatives of other genera exhibited substantially higher diversity levels and more pronounced divergence across large portions of the genome.

Regional variation in nucleotide diversity was further examined in the comparison between PMV and representative *Macochavirus* sequences. In the first half of the genome, corresponding primarily to the Rep‐encoding region (positions 14–1195), nucleotide diversity values were relatively elevated, with *π* values ranging approximately from 0.04 to 0.11, indicating greater sequence divergence in this region. In contrast, nucleotide diversity was noticeably lower in the middle‐to‐late portion of the genome corresponding to the Cap‐encoding region (positions 1404–2560), where *π* values ranged approximately from 0.03 to 0.07. Consequently, the diversity profile in the Cap‐encoding region appeared to be more conserved compared with that observed in the Rep‐encoding region.

Overall, these genome‐wide nucleotide diversity patterns are consistent with the phylogenetic analyses and further support the close evolutionary relationship between the proposed PMV lineage and representative *Macochavirus* sequences.

## 4. Discussion

In this study, metagenomic sequencing of fecal samples from diarrheic pigs in China led to the identification of 10 complete circular single‐stranded DNA viral genomes. Phylogenetic and comparative analyses consistently demonstrated that these viruses are not related to the known PCV1–PCV4 but instead belong to the family Pecoviridae and are closely associated with previously reported FSfaCV/PCV5‐like viruses [16, 28]. Based on multiple complementary lines of evidence, including phylogenetic inference, sequence identity, genetic distance, haplotype network, and nucleotide diversity analyses, these viruses, together with FSfaCV/PCV5‐related sequences, are proposed to represent a distinct porcine lineage within the genus *Macochavirus*, designated PMV.

Although PMV genomes were detected in pigs exhibiting clinical signs of diarrhea, no direct evidence currently supports a causal relationship between these viruses and disease. Metagenomic analysis of the positive samples revealed the presence of multiple other viral and bacterial agents, including porcine bocavirus, porcine serum‐associated circular virus, cycloviruses, porcine parvovirus type 5, *Escherichia coli*, Klebsiella spp, and Streptococcus spp. Moreover, the detection of PMV genomes in fecal samples alone does not necessarily indicate active infection or replication in pigs, as viral nucleic acids may originate from dietary or environmental exposure and pass transiently through the gastrointestinal tract. This consideration is particularly relevant for cressdnaviruses, which have been identified across a wide range of hosts and environmental sources. Nevertheless, recent studies have provided evidence supporting an association between the FSfaCV/PCV5 lineage and swine [16]. For example, viral DNA has been detected at a high prevalence in clinical swine samples, with viral load positively correlated with the severity of pathological lesions, and viral proteins have been identified in intestinal tissues exhibiting histopathological changes [16]. Although these findings do not conclusively establish pathogenicity or tissue tropism, they argue against the possibility that members of this viral lineage represent only dietary contaminants. Therefore, the pathogenic potential, natural host range, tissue tropism, and biological significance of PMV remain unclear and warrant further investigation through controlled virological, epidemiological, and experimental studies.

Cressdnaviruses have been increasingly reported in swine in recent years [7, 8], highlighting their extensive diversity and complex biological roles. Among them, PCV2 is a well‐established pathogen associated with PCV‐associated disease [15], demonstrating that certain cressdnaviruses can have significant impacts on animal health and the livestock industry. In contrast, many other cressdnaviruses detected in pigs, including porcine serum‐associated circular viruses and cycloviruses [8, 29], have uncertain pathogenic roles and are frequently identified in both healthy and diseased animals. In addition, our previous study identified a novel gemykibivirus in a diarrheic pig in China using metagenomic analysis, further illustrating the diversity of cressdnaviruses in swine [30]. Collectively, these findings indicate that pigs harbor a complex and dynamic community of cressdnaviruses, ranging from well‐characterized pathogens to poorly understood viral lineages. Within this broader context, the identification of PMV adds a new component to the porcine cressdnavirus virome.

Within the family Pecoviridae, the taxonomic status of FSfaCV/PCV5‐related viruses has remained unresolved [16, 31], and these viruses have generally been considered unclassified members. The present study provides convergent evidence that these viruses, together with the newly identified porcine genomes, form a coherent evolutionary lineage that is embedded within the diversity of the genus *Macochavirus*. Although the nucleotide identities and genetic distances between PMV and previously reported *Macochavirus* reference sequences indicate a degree of divergence, these values fall within the range of intra‐genus variation. These results support the classification of PMV within *Macochavirus* and refine the genus‐level taxonomy of FSfaCV/PCV5‐related viruses. Importantly, the identification of PMV expands the currently recognized diversity of *Macochavirus* by defining a distinct porcine‐associated lineage.

An additional observation from this study is the differential evolutionary patterns observed between the Rep‐ and Cap‐encoding regions. Phylogenetic analyses and nucleotide diversity profiling consistently showed that the Rep‐encoding region exhibits higher levels of sequence divergence compared with the Cap‐encoding region. Correspondingly, the Rep‐based phylogeny provided clearer resolution at the genus level, whereas the Cap‐based phylogeny revealed a closer association between PMV and representative *Macochavirus* sequences. These findings indicate that different genomic regions contribute unequally to phylogenetic inference and underscore the importance of incorporating multiple genomic markers when evaluating the evolutionary relationships of cressdnaviruses.

The identification of PMV in pigs from six geographically distinct provinces in China expands the currently recognized distribution of the FSfaCV/PCV5‐related lineage [16, 28, 31]. Together with previous reports from Japan, New Zealand, and China, these findings suggest that this viral lineage may be more widely distributed than currently appreciated. Continued surveillance and genomic characterization will facilitate a better understanding of its geographic distribution, host range, and evolutionary history. In addition, this study is based primarily on metagenomic sequencing and comparative genomic analyses, and no virus isolation or functional characterization was performed. Future studies incorporating broader surveillance, virus isolation, and experimental validation will be essential to better understand the epidemiology, host interactions, and biological properties of PMV.

In summary, this study identifies and characterizes a novel porcine‐associated lineage of cressdnaviruses and provides multiple lines of evidence supporting its classification within the genus *Macochavirus*. The designation of PMV not only resolves the taxonomic ambiguity surrounding FSfaCV/PCV5‐related viruses but also expands the current understanding of viral diversity and evolution within the family Pecoviridae.

## 5. Conclusions

In conclusion, multiple lines of evidence consistently support that the viral genomes identified in this study, together with FSfaCV/PCV5‐related viruses, form a genetically distinct and stable lineage within the family Pecoviridae. The strong concordance among these independent analytical approaches enhances the robustness of this taxonomic inference and clearly distinguishes this lineage from other members of the family.

Comparative analyses of the Rep and Cap proteins further revealed heterogeneous evolutionary constraints across different genomic regions, highlighting the importance of incorporating multigene evidence in the classification of cressdnaviruses. The findings of this study provide new insights into the evolutionary relationships and taxonomic framework of porcine cressdnaviruses and contribute to a more comprehensive understanding of their genetic diversity within swine populations.

## Author Contributions

Wenqiang Wang, Xiangdong Li, Kegong Tian, and Lin Yuan conceived and designed the study. Liping Jiang, Wenqiang Wang, and Tingting Niu performed the experiments, analyzed the data, and drafted the manuscript. Miaojie Zhang, Lulu Chen, and Xiaoye Jia contributed to revise the manuscript.

## Funding

This work was supported by the National Key Research and Development Program of China (Grant 2023YFD1800500), the National Natural Science Foundation of China (Grant 32500538), the 111 Project (Grant D18007), and the Priority Academic Program Development of Jiangsu Higher Education Institutions (PAPD).

## Disclosure

All authors have read and approved the final manuscript.

## Ethics Statement

This study involved the analyses of viral sequences obtained from metagenomic sequencing data and publicly available genomic datasets retrieved from established databases. No human participants, animals, or identifiable personal information were directly involved, and no experimental procedures were performed on living subjects in this study. Therefore, ethical approval and informed consent were not required.

## Conflicts of Interest

The authors declare no conflicts of interest.

## Data Availability

The original contributions presented in this study are included in the article. Further inquiries can be directed to the corresponding authors.
